# Climate change and population: Demographic perspectives on the 21st century’s defining challenge

**DOI:** 10.1553/p-nfjc-z82h

**Published:** 2024-12-15

**Authors:** Roman Hoffmann, Liliana Andriano, Erich Striessnig, Tobias Rüttenauer, Marion Borderon, Kathryn Grace

**Affiliations:** 1 International Institute for Applied Systems Analysis (IIASA), Wittgenstein Centre for Demography and Global Human Capital (IIASA, VID/OeAW, University of Vienna), Laxenburg, Austria; 2 Department of Social Statistics and Demography, University of Southampton, Southampton, UK; 3 Department of Demography, University of Vienna, Wittgenstein Centre for Demography and Global Human Capital (IIASA, VID/OeAW, University of Vienna), Vienna, Austria; 4 Social Research Institute, University College London, London, UK; 5 Department of Geography and Regional Research, University of Vienna, Vienna, Austria; 6 Minesota Population Center, University of Minesota, Minneapolis, USA

**Keywords:** Climate change, Climate impacts, Demography, Population, Environment

## Abstract

Climate change represents one of the most pressing challenges for societies in the 21st century. This special issue of the Vienna Yearbook of Population Research (VYPR) brings together interdisciplinary contributions from 51 authors to explore the demographic dimensions of climate change. In many ways, human populations are at the center of the current climate crisis. On the one hand, anthropogenic forces are responsible for the unprecedented changes in the climate system that are currently being observed. It is the burning of fossil fuels that has significantly increased greenhouse gas concentrations, driving global warming and altering natural climate patterns. On the other hand, human populations are also profoundly affected by these changes, as they are facing increased risks from extreme weather events, rising sea levels and shifting ecosystems, which, in turn, impact livelihoods, food and water security, and health and well-being. This special issue provides a comprehensive overview of both the role of population as a driving force of climate change and the significance of its impacts in the areas of health and mortality, migration, and fertility and reproductive behaviors. In addition to 10 research articles, the special issue features seven debate articles by leading scholars, who provide reflections on the climate-population nexus and the role of demographic science in climate change mitigation. Demography offers a wide range of perspectives and methodological tools to understand and address the climate-population nexus, including in the areas of health and population data, mathematical and statistical modeling, and projections. We advocate for a holistic research perspective that incorporates issues related to increasing climate risks into demographic thinking, and vice versa. A thorough understanding of the intricate relationship between populations, population dynamics and climate change is necessary for the development of effective and equitable mitigation and adaptation strategies that address both global and local challenges over time.

## Introduction

Climate change is increasingly affecting communities worldwide, posing a significant threat to human health and well-being (IPCC, 2022). Climate impacts can take various forms. Threats such as rising temperatures, shifting precipitation patterns, pervasive drought conditions, rising sea levels and glacial retreat evolve over longer time periods and alter ecosystems, agricultural productivity, livelihood opportunities and food and water availability. Simultaneously, rapid-onset events such as severe storms, flooding and wildfires are becoming more frequent, unpredictable and intense because of climate change, posing immediate and longer-term threats to those affected.

Importantly, the impacts of climate change are not distributed equally across time, space and different population subgroups. For example, generations born today are far more likely than their parents and grandparents to experience extreme climatic events in their lifetimes (Thiery et al., 2021). Moreover, the impacts of climate change will be more serious for places or people who are highly exposed to climate hazards and vulnerable to their effects, for example, because of poor health, low levels of education, poverty, or social and political marginalization. These factors can limit the capacity and the resources of individuals and communities to cope with and adapt to changes, and can thus exacerbate the impacts they experience.

Human populations are not only affected by the impacts of climate change, but are also its primary driver through the emissions they produce and other activities they engage in that contribute to environmental degradation. Over the last 150 years, human activities, particularly the emission of greenhouse gases through economic production, transportation, heating and other processes, have led to the warming of the atmosphere. While anthropogenic climate change is influenced by the number of people living on Earth, it is mainly caused by people’s affluence levels and their consumption and lifestyle patterns, which vary widely across the planet (Merchant, 2021; Muttarak, 2021). This results in deep injustices, as those who are the most affected by the climate crisis are typically those who have contributed the least to it (Zimm et al., 2024).

A growing interdisciplinary research community is seeking to improve our understanding of the myriad of interactions between humans, the environment and climate change by examining both the causes and the consequences of these complex relationships. Demography plays a key role in explaining these processes (Lutz et al., 2005; Muttarak, 2021). Demographic factors such as population size, composition and spatial distribution significantly influence both the causes and the consequences of climate change. For example, when populations are growing, the demand for resources and energy increases, potentially contributing to higher emissions. At the same time, population characteristics such as age and education structure can play an important role in explaining lifestyle and consumption differences, and hence the overall environmental footprint of a country.

Demographic factors and processes are also highly influential in determining differential vulnerabilities to climatic stress (Thomas et al., 2019). For example, children and older individuals are more susceptible to health risks associated with climate change due to their physiological characteristics, general health condition and reduced ability to cope with extreme events. Climate hazards can also disrupt the availability and accessibility of health services, for example, by destroying infrastructure or obstructing access routes. Such disruptions can be especially problematic in the context of reproductive health and rights, with pregnant women and mothers facing particular challenges and heightened vulnerability due to their specific health needs.

Migration is another important demographic process that can lead to populations moving to areas less equipped to handle environmental stress, including informal settlements, which often lack the infrastructure, sanitation and healthcare services needed to adequately respond to extreme events (Hoffmann and Muttarak, 2021). Increased urbanization in many parts of the world concentrates populations in areas that may be particularly vulnerable to climate-related hazards, further complicating climate change mitigation and adaptation efforts. On the other hand, this increased concentration can contribute to sustainability transitions by enhancing people’s well-being without exacerbating structural inequalities or placing additional strain on environmental resources (Adger et al., 2024).

To highlight the role of population in the causes and the consequences of climate change, this special issue of the Vienna Yearbook of Population Research (VYPR) brings together perspectives from 51 authors. The volume consists of 10 research articles covering issues related to the role of population in climate change mitigation, the impact of climatic factors on health and mortality, migration and reproductive behavior, and differential vulnerabilities and adaptive capacities, among others. These contributions employ diverse methodologies from the demographic toolkit and provide evidence from cases across various regions of the world.

In addition, the special issue includes seven Debate articles written by leading scholars in the field who discuss the importance of population and demographic trends for climate change mitigation, and how population policies can be used to tackle climate change. This unique collection of critical reflections complements the empirical articles, and is meant to provide readers with an overview of the current discussions, as well as the authors’ positions in these discussions, against the background of their research and the broader literature. The Debate articles discuss the significant role humans play in affecting the climate, albeit to greatly varying degrees, emphasizing the need for a nuanced perspective on populations’ contributions to global warming. They also underscore the importance of demographic data and rigorous evidence in supporting climate mitigation and adaptation efforts, while highlighting the urgent need for interdisciplinary collaboration and coordinated policy actions to understand and address the profound injustices of the climate crisis.

This introductory article first provides an overview of the climate-population nexus. In the following section, key insights from the literature on the relevance of population as a driver of climate change are presented. Next, the major impacts of climate change on population outcomes and dynamics are discussed in three sub-sections, with a particular focus on climate impacts on health and mortality, migration, and reproductive behaviors and fertility outcomes. As the impacts of climate change are expected to become even more detrimental in the future, the following section highlights the role of population projections and integrated forward-looking perspectives in capturing the full scope of the climate-population nexus and its implications today and in the future. Building on the contributions to this volume, we argue in the final section of this introductory article that a holistic demographic perspective is needed to understand the complex interplay between population dynamics and climate change, and to inform comprehensive policies and adaptive strategies aimed at mitigating its impacts.

## The climate-population nexus

**Figure 1.**
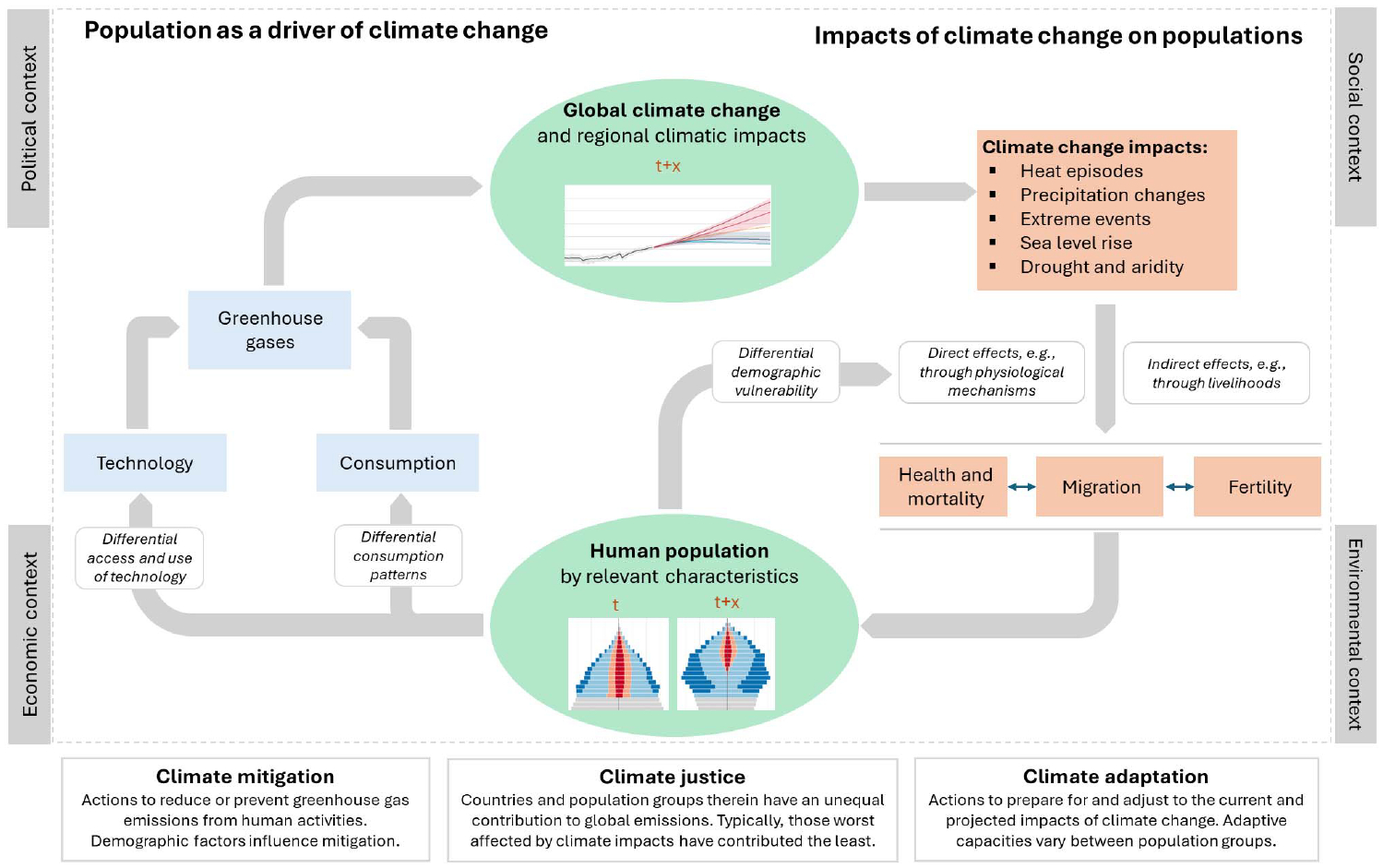
The climate-population nexus. The figure shows the multiple ways in which humans influence the climate system and how climate change affects populations by influencing health, migration and fertility outcomes. Population groups contribute to these processes differently, and are affected by them differently. Source: Authors’ own illustration, elaborating on the framework presented in Lutz and Muttarak (2017) with permission from Springer Nature.

Human populations are at the center of climate change and its impacts (Lutz et al., 2005). On the one hand, anthropogenic forces are responsible for the unprecedented changes in the climate system that are currently being observed. On the other hand, humans are directly affected by the resulting impacts. This bidirectional relationship, depicted in Figure 1, highlights the relevance of population as a driver of global climate change and its local impacts (left), and the multiple possible effects of changes in climatic conditions on population outcomes and the resulting dynamics (right). These processes are shaped by the economic, political, social and environmental context, which can influence mitigation and adaptation options, resource allocation, and the ability of people to withstand and cope with climatic impacts.

Humans influence the climate primarily through the emission of greenhouse gases related to various activities, such as burning fossil fuels for energy and industrial production processes, agriculture, transportation and deforestation. Technological advancements can play an important role in mitigating these emissions over the long term. However, whether these technologies can reduce emissions over the short and medium term continues to be debated. To combat climate change starting immediately, decarbonization as well as shifts in consumption habits and lifestyles are urgently required, especially in wealthier countries with high (historic) emission levels.

The accumulation of greenhouse gas emissions in the atmosphere causes global warming. Since the 19th century, global temperatures have increased by nearly 1.2 °C, with low latitude regions warming up particularly fast. This has led to a range of changes in local climatic conditions and extreme events, which are commonly referred to as climate change impacts. These include risks due to heat episodes, changes and increased variability in precipitation patterns, extreme weather events such as storms and flooding, sea level rise and increased drought and aridity.

These climate change impacts have been affecting communities worldwide. Here, direct and indirect channels of influence can be distinguished. Climate change affects humans directly by posing existential threats to their health and well-being in the form of shocks and physiological impacts. In addition, climate change exerts indirect effects on populations through its impacts on livelihoods, food and water security, social stability and peace. Both direct and indirect effects can influence key drivers of population change, including population health and mortality, migration and fertility patterns, with implications for the size and the composition of future populations in different parts of the world. These demographic changes will, in turn, determine the exposure and the vulnerabilities of future populations to climate events.

Both the responsibility for climate change and the consequences of climate change are not equally distributed. High-income countries, which are historically the largest emitters of greenhouse gases due to their early industrialization and high consumption levels, bear a significant responsibility for the current state of the climate. However, it is often the low-income countries and the most vulnerable populations within all countries that bear the brunt of the impacts. This imbalance highlights the profound injustice of climate change, as those who have contributed the least to the problem are often the most severely impacted, and thus face greater challenges in coping with and recovering from its effects.

Differential vulnerabilities are intrinsically linked to inequalities in societies, and to challenges in achieving just and inclusive development both within and across countries. At the same time, climate change also creates significant intergenerational injustices, as future generations will inherit the long-term consequences of climate change without having contributed to it. Thus, recognizing population heterogeneity is important for understanding both the causes and the consequences of climate change. Different population subgroups – both in the present and in the future – can have vastly different mitigation and adaptation options, while experiencing varying levels of exposure and vulnerability to climate change impacts.

How climate change will affect future populations and demographic trends over the short and the long term, and the role of global socioeconomic development in these processes, are the subjects of ongoing research. Using projection methods, demographers and population scientists are exploring the interplay of the different factors, and are developing scenarios describing potential population trajectories. Depending on the decisions taken by societies today, these scenarios can paint a more positive or a more negative picture. Policy interventions across multiple domains can play an important role in shaping the trajectories of population and socioeconomic developments, and in mitigating the risks associated with climate change in just and inclusive ways.

## Is population a driving force of climate change?

Population and population growth have long been discussed as drivers of climate change. Influential scholars such as Thomas Malthus, Garret Hardin and Paul Ehrlich have argued that overpopulation is the single most important and critical driver of environmental degradation and climate change (Bongaarts, 2023; van Dalen and Henkens, 2021). Although the topic had been discussed earlier, the overpopulation debate gained momentum during a time when global population was growing, peaking at an annual rate of 2.3% in 1963 (UN DESA, 2019). In their influential work, Hardin and Ehrlich used apocalyptic and unrealistic projections of future environmental conditions and food security to argue for drastic population control measures (Ehrlich, 1968; Hardin, 1968).

The efficacy and moral implications of such population control measures have been discussed by scholars. Referring to the neo-Malthusian population discussion, Pachauri argues in her Debate article (this volume) that the carbon footprint of childbearing decisions can be considered alongside the carbon footprint of any other consumption choice. However, the estimated carbon footprints of children and the assumptions about future consumption behaviors vary across studies. Moreover, Pachauri’s article underscores the importance of free choice, human rights and social justice considerations in climate change mitigation efforts, emphasizing that the environmental sustainability of individual consumption and fertility behaviors needs to be addressed in an ethical and just way.

Since the 1960s, population growth has declined, and increases in food production have out-paced population growth (Lam, 2023). Recent projections suggest that the global population will peak in the second half of the 21st century before starting to decline (Raftery et al., 2012; UN DESA, 2024). However, the discussion among demographers as well as the wider public about population as a major driver of climate change, and the potential need for population control measures, has persisted (van Dalen and Henkens, 2021).

The IPAT model (Liddle, 2014) provides a framework for formalizing the relationship between population and environmental impacts: it estimates environmental impacts (I) as a product of population (P), affluence (A) and technology (T). In her Debate article, Muttarak (in this volume) critiques this simplistic approach and discusses how it has been revised and refined to better account for spatial and socioeconomic heterogeneities, as well as other demographic characteristics, such as age, sex and education.

Importantly, the IPAT framework overlooks the reality that the benefits of technological developments are stratified along socioeconomic lines, with wealthier individuals within countries and populations in the Global North having better access to efficient technologies across various domains, such as housing, transportation and electricity (Vaishnav, 2023). In sub-Saharan Africa, for example, only 43% of the population currently have access to electricity, and the geographic coverage of electrical infrastructure is likely to remain limited over the coming decades (Dalla Longa and van der Zwaan, 2021). This disparity in technological access hampers climate mitigation efforts, as less affluent regions struggle to achieve climate neutrality.

Focusing solely on population numbers oversimplifies the complex dynamics of how populations affect climate change. As Adamo argues in her Debate article (this volume), population migration and urban-rural flows will increasingly influence the climate impacts experienced by different geographic regions and the potential for climate change mitigation in the future. The exclusive focus on population numbers also overlooks the significance of individual consumption behaviors. The consumption of animal-based products, individual travel choices and heating practices, among other behaviors, play a significant role in shaping the nexus between society and climate change impacts (Bruderer Enzler and Diekmann, 2019; Jorgenson et al., 2019; Rüttenauer, 2023).

According to recent estimates, two-thirds of global greenhouse gas emissions are linked to individual consumption, with the largest impacts coming from mobility and transportation, housing and nutrition (Ivanova et al., 2020). The carbon-intensity of people’s behavior follows a strong social gradient. On average, well-educated and higher-income households tend to have higher per person greenhouse gas emissions. In 2019, the richest 1% of the world’s population accounted for an estimated 16% of global carbon emissions – equivalent to the combined emissions of the poorest 66% of the world’s population, or five billion people (Khalfan et al., 2023). Women and older individuals generally have a smaller ecological footprint, and there is a complex relationship between urbanization and emissions (Liddle, 2014). This demographic and economic gradient in greenhouse gas emissions adds yet another dimension of social (in)justice (Bruderer Enzler and Diekmann, 2019).

Education is a crucial aspect of population heterogeneity that significantly influences climate change and its mitigation. Increased education is typically associated with a reduction in fertility rates, as it empowers women while simultaneously increasing economic activities (O’Neill et al., 2020). The extent to which these increasing economic activities impact climate change hinges on technological developments. Moreover, population dynamics often affect climate change through both direct and indirect pathways. In addition to influencing climate change via fertility behavior, education enhances the adaptive capacity of societies, thereby mitigating the potential negative impacts of extreme climatic events (Lutz et al., 2014).

Public opinions and attitudes toward climate changes are another important factor. Climate change beliefs and general values have been studied extensively as drivers of individual pro-environmental action (Steg, 2023). However, despite the growing awareness of climate change and the increasing support for green policies (Hoffmann et al., 2022a), climate-relevant behaviors and habits remain surprisingly resistant to change (Hoffmann et al., 2024; Rüttenauer, 2023; van Valkengoed and Steg, 2019). According to the problem of collective action (Olson, 1971), every individual has an incentive to freeride, waiting for others to undertake costly mitigation behaviors (Ostrom, 1998). In the end, everyone contributes less than would be optimal, in a situation akin to coordinating the cleaning of dirty dishes in a shared apartment with eight billion people. To address this problem and to effectively harness the changing attitudes of populations, societies need to redesign the incentive structures with monetary and non-monetary incentives such as green defaults (Liebe et al., 2021), and to implement carefully designed policy measures (Fesenfeld et al., 2022). Redistributive elements and the perceived fairness of these policy instruments play a vital role in enhancing public acceptance (Beiser-McGrath et al., 2023; Bergquist et al., 2022).

Population dynamics influence climate change through various complex pathways beyond sheer population numbers. Migration patterns, urbanization, education and socioeconomic factors shape the impact of populations on climate change – though precisely how populations affect climate change can vary depending on the specific context, the availability of technologies and individual consumption behaviors. Access to green technologies and their contributions to climate change through individual consumption choices are significantly influenced by economic resources, underscoring the importance of the issue of social justice in the relationship between population and climate change.

## Understanding climate change impacts on populations

Climate change can affect demographic processes through its impacts on health and mortality, migration and fertility. Articles in this special issue document the various ways in which populations are affected, showing highly diverse effects across regional contexts and population subgroups.

### Health and mortality

Climate change can significantly affect health outcomes, leading to a variety of problems such as heat-related illnesses, the spread of infectious diseases, food insecurity, water-related illnesses and increases in injuries and fatalities. These effects are generally not uniformly distributed across all segments of the population, highlighting significant disparities in levels of vulnerability and exposure (Muttarak, 2021). For example, older individuals are particularly susceptible to extreme temperatures – including both heatwaves and cold spells – due to their reduced ability to thermoregulate. Similarly, newborns and children face increased risks from malnutrition and infectious diseases, both of which are exacerbated by environmental changes such as rainfall anomalies or heatwaves. These conditions can lead to higher rates of underweight and stunting and lower birthweights, which may, in turn, result in long-term health issues (Andriano, 2023; Davenport et al., 2020; Deschênes et al., 2009; Dimitrova and Muttarak, 2020; Grace et al., 2015, 2021; Kumar et al., 2016; Rocha and Soares, 2015).

The health impacts of extreme temperatures vary based not only on individual characteristics, but also on environmental factors. Zanasi and Conte Keivabu (this volume) have investigated the impact of extreme temperatures on hospitalizations and cardiovascular diseases based on data from the Survey of Health, Ageing and Retirement in Europe (SHARE). They find that extremely cold temperatures have a larger impact on health than extreme heat in all regions of Europe, while extreme heat increases the risk of hospitalization in the warmest regions only. Zanasi and Conte Keivabu also find that older and lower educated individuals are particularly vulnerable to temperature-related health impacts.

In addition to looking at regional differences, examining urban-rural disparities and settlement patterns is important for understanding climate change impacts on health. Maksimenko et al. (this volume) have explored spatial heterogeneity in mortality risks during the 2010 heatwave in European Russia. They find that urban areas had a 52% spike in age-standardized mortality rates, while the impact in rural settings was significantly lower. Despite having better healthcare infrastructure, urban areas often suffer from “heat island” effects exacerbated by dense construction and inadequate ventilation, which intensify the effects of high temperatures and lead to a steep “heat slope” between heat stress and settlement size.

Gender, preexisting health conditions and socioeconomic status further compound the health risks of extreme temperatures (Poumadère et al., 2005). With a specific focus on gender, research from Arsenović et al. (this volume) on the distinct health impacts on men and women underscores the intersecting effects of biological and social determinants of health. Increased urban outdoor thermal conditions correlate with an increased cumulative relative risk of cardiovascular hospital admissions for males, and an increased cumulative relative risk of respiratory hospital admissions for females. This discrepancy might reflect gender-specific differences in occupational exposures, healthcare access or underlying health conditions.

Disparities in vulnerabilities also vary based on the societal context. In settings where gender discrimination is rampant, the effects of climate change can exacerbate existing inequalities. For example, girls in India face a higher risk of stunting due to rainfall anomalies than boys, reflecting gender-based discrimination in feeding practices and general patterns of neglect in financially strained households (Dimitrova and Muttarak, 2020). Conversely, male newborns are highly vulnerable to heatwave exposure in sub-Saharan Africa, suggesting a male disadvantage at birth due to heat-induced stress (Andriano, 2023).

In addition to the direct effects of thermal and other climatic stress on health outcomes, temperatures can indirectly worsen pre-existing health risks by increasing metabolic stress and reducing the body’s ability to cope with infections and nutritional deficits, potentially leading to severe or fatal outcomes. Junkka and Hiltunen (this volume) have investigated how temperature variations affected vulnerability to water- and foodborne infectious diseases among infants based on register data from Sweden between 1868 and 1892. They show that while temperatures did not affect the impact of airborne infectious diseases, hot temperatures significantly increased the impact of water- and foodborne infectious diseases on infant mortality.

Other indirect channels can contribute to the total effects of climate change on health and mortality. For example, the economic losses associated with climate change increasingly harm livelihoods, limit resilience and restrict the funds available for climate adaptation. Economic losses from extreme weather events have increased sharply over the past decade. In 2022, such events led to estimated losses of US$264 billion due to infrastructure damage and estimated income losses of $863 billion due to reduced labor productivity (Romanello et al., 2023). These losses and damages can have significant detrimental effects on health outcomes by exacerbating poverty, undermining public health infrastructure, reducing access to healthcare and increasing levels of insecurity, all of which can contribute to higher morbidity and mortality rates.

Despite these alarming trends, adaptation efforts and technological progress, like the spread of air conditioning and early warning systems, have led to a decrease in heat- and cold-related deaths in high-income countries (Achebak et al., 2019; Barreca et al., 2016). Whether these trends will extend to other climatic shocks, and whether such solutions will be scalable and effective in low- and middle-income countries over the short term, remains uncertain, largely due to economic, infrastructural and policy constraints.

### Migration

The question of whether and to what extent climate change influences migration patterns has received considerable attention in the public and scientific debates. While various studies have shown that environmental hazards can be a relevant driver of mobility, the direction and the size of the impact vary depending on the local context, the characteristics of the affected population and the nature of the impacts experienced (Call et al., 2017). Indeed, while some studies have reported significant increases in migration due to climate change-induced crises such as extreme weather events and sea level rise, others have found no or a negative effect (Beine and Jeusette, 2019; Borderon et al., 2019; Hoffmann et al., 2020; Šedová et al., 2021).

Climate change and related impacts do not automatically lead to more mobility, as they can also reduce people’s ability and willingness to move, thus contributing to increased immobility (Carling, 2002; Zickgraf, 2018). In some situations, this can result in populations becoming trapped during crisis events, which can lead to heightened vulnerability and a potential exacerbation of the losses and damages they experience. Importantly, the risks and the impacts of climate change may not affect all population groups and their mobility equally (Muttarak and Lutz, 2014; Thomas et al., 2019). While some people may be willing and able to leave, others may prefer or be forced to stay, resulting in differential mobility patterns within a larger population (Adams, 2016; Upadhyay et al., 2015).

When climatic factors influence migration patterns, they typically have a larger impact on short-distance internal mobility than on long-distance cross-border migration (Hoffmann et al., 2020). In some cases, climatic changes can also alter the nature of existing migration patterns within a region, such as when nomadic households modify their pastoralist routes or when the timing and the necessity of seasonal mobility are affected. This diversity of impacts underscores the importance of recognizing the wide spectrum of climate-induced mobilities, including micro-mobilities (Boas et al., 2022; Wyss and Dahinden, 2022).

Climatic factors do not affect migration in isolation, but in close interplay with other factors and drivers (Black et al., 2011). Local social and economic conditions can influence the extent to which households or individuals within households are exposed and vulnerable to hazards, and can ultimately determine their aspirations and capabilities to migrate (de Haas, 2021). In this context, previous work has emphasized the importance of the agricultural channel in mediating the impacts of climate change. Agriculturally dependent households are particularly vulnerable to climatic hazards, and are more likely to change their mobility patterns in response (Biella et al., 2022; Hoffmann et al., 2022b).

In addition, wealth levels can moderate the climate-migration relationship. Often it is not the poorest parts of a population who become mobile, but rather the middle-income groups who possess more capacities and resources (including immaterial resources such as information or networks) and face fewer mobility constraints. Demographic factors at the household level further determine who becomes mobile, including the age, gender and health of the household members, and their role in the family (Hunter et al., 2015; Lama et al., 2021).

Importantly, the relationship between climatic stress and migration is shaped by the local context. In their study on Bangladesh, Donato et al. (this volume) show that the relationship between erosion and the likelihood of making a first domestic or international trip is moderated by livelihood type and land ownership. With worsening environmental conditions, the odds that non-agricultural, non-landowning household heads will make a first domestic trip rise, and the odds that landowners working in agriculture will make a first domestic trip decline. These differential effects suggest that being tied to land is another important factor in explaining which households will become mobile under environmental stress. In line with this finding, a growing literature highlights the important role of place attachment and local networks in shaping mobility and immobility patterns (Farbotko et al., 2020).

Conflict and sociopolitical instability are important factors in migration trends, both as channels of influence through which climatic factors influence migration, and as important moderators exacerbating the consequences of climatic stress experienced by households (Watson et al., 2023). Indeed, previous studies have highlighted how climatic factors can lead to increased conflict in sociopolitical environments characterized by instability, which can, in turn, force people to leave their homes (Abel et al., 2019). Two studies in this special issue further address the climate-conflict-migration nexus.

Focusing on Columbia as a case study and using detailed municipality-level data, Fenz et al. (this volume) show how drought has affected conflict risks and internal migration in the country. Their results indicate a positive relationship between drought severity and conflict as well as between conflict and human mobility, which suggests that conflict can be an indirect mechanism connecting climatic factors and migration. They also find that droughts tend to have a stronger effect on smaller conflicts and in areas that have not experienced another conflict in previous years.

Abdel Ghany (this volume) has used data on asylum applications to study the relationship between temperature anomalies, conflict risks and forced international migration in the Middle East and North Africa. While she documents a weak link between the considered climatic factors and conflict, she finds no significant association between temperature extremes and migration, underlining the importance of accounting for contextual factors in the analysis of migration patterns. However, she also shows that conflict is a strong predictor of international asylum flows from the region.

In addition to research exploring the effects of climatic factors on migration, another strand of research has focused on the consequences of migration, for example in the context of climate change adaptation (Black et al., 2011; McLeman and Hunter, 2010). Vinke et al. (2020) find that while migration can serve as an effective adaptation strategy for some groups in specific circumstances, it can also exacerbate people’s vulnerabilities and initiate a cycle of poverty, thereby diminishing their ability to adapt (Sakdapolrak et al., 2024). Moreover, the adaptive potential of migration could be limited by different mechanisms, such as simultaneous exposure: i.e., geographically distant places may face risks simultaneously due to the global or systemic character or the multiplicity of crises (Sakdapolrak et al., 2024).

In the context of climate change, migration can also have important implications for climate mitigation efforts. Few studies have considered this aspect with the exception of research on daily mobilities, transportation and tourism. In her Debate article, Adamo (this volume) highlights that “climate change mitigation strategies and actions need to take into account their potential interactions with population mobility because it is a key component of population growth, population distribution and urbanization trends, as well as a potential contributor to behavioral change.” She also points to the need for further research in this important area to improve our understanding of how climate-induced migration intersects with mitigation efforts, and of how policies can be designed to effectively address both migration dynamics and climate change mitigation goals.

### Fertility

Climate and weather conditions are related to fertility behaviors and outcomes through biological and behavioral pathways (Barreca et al., 2018; Brooks et al., 2023; Grace, 2017). For decades, demographers have explored the ways that environmental conditions – including seasonal variability and disease environments – have shaped women’s reproductive biology, behaviors and outcomes through their effects on, for example, food security and seasonal labor demands (Bongaarts, 1980; Lee and Kramer, 2002; Merchant, 2021; Panter-Brick, 1996). Now, in a scientific and a popular context where concerns about anthropogenic climate change are rising, scientific research exploring the impacts of climate change on people’s lives, including on women’s reproductive health, has grown rapidly.

Demographers have explored a range of different linkages that connect climate change to reproductive health and fertility, often focusing on some combination of the following three mechanisms: (1) direct physiological impacts of meteorological extremes on fecundity, the frequency of sexual intercourse, pregnancy and birth outcomes, and reproductive health (Segal and Giudice, 2022); (2) seasonal variability in resources and resource management with short-term impacts on contraceptive use, fecundity, birth timing and pregnancy outcomes; and (3) indirect impacts of climate change concerns and climate anxiety on fertility ideation and subsequent realization.

The first two mechanisms are often explored in the context of low-income countries or poor communities, where impoverished individuals and communities are facing changing environmental conditions with few formal institutional buffers that could help to reduce the impacts of these changes on, for example, their health, social security and food systems. The third mechanism is mostly studied in the context of wealthier countries, where prospective parents are supposedly more worried about the future impacts of climate change on their hypothetical offspring, and/or where the concept of “environmental stewardship” entails a responsibility for slowing climate change through reduced population growth and resource use (Powdthavee et al., 2024; Schneider-Mayerson and Leong, 2020).

Yet concerns about climate change and its potential impacts on quality of life are by no means expressed in developed countries only, as shown in this special issue by Brooks (this volume). Looking at an integrated dataset of Demographic and Health Surveys (DHS) from five African countries, she finds that temperature positively affects people’s fertility ideals, particularly among those who live in the arid regions of the Sahel and those who do not have land of their own. She also shows that while men’s ideal number of children is more robust to changes in climatic conditions than that of women, men express a stronger preference for sons in hotter climates. All in all, these results emphasize the important role of reproductive decisions in responses to growing uncertainty around climate change.

## Projecting future demographic change and its implications

Demographic projections are an important instrument in the demographic toolkit, both for enabling near-term public planning and assessing the long-term consequences of future anthropogenic global warming, biodiversity loss and environmental conservation. While the accuracy of population forecasts can only be evaluated ex post, demographers strive to base their future assumptions on the best available empirical evidence and to feed these assumptions into state-of-the-art models of population change, which are being applied to increasingly smaller spatial and temporal scales (Wilson et al., 2021).

Projections are particularly relevant for informing policymakers about the potential characteristics and dynamics of future populations. Madise et al. argue in their Debate article (this volume) that the future developments in the most populous and fastest growing countries will be particularly important. Despite their high absolute emissions, many countries in the Global South have very low per capita emissions. The future consumption patterns and economic development pathways in poor but fast-growing countries will thus become decisive variables in the mitigation of climate change. Madise et al. argue that “development aid must be available to ensure that poor countries can address their food security and energy needs with sustainable means” (this volume). Thus, current mitigation efforts should be viewed from a temporal perspective that considers historical inequalities and future population dynamics.

With regard to population-environment interactions, demographic projections help to anticipate future demand for essential resources, such as food, water, energy or land. This is highlighted by the contribution of Soltani et al. (this volume), who have studied the question of water scarcity in Iran under different fertility scenarios within a water-population system dynamics model. As Iran is situated in semi-arid to arid climate zones, water security has become an increasing concern in the country, especially given that the demographic transition has led to a rapid increase in population. By improving our understanding of future population trends and the spatial distribution of populations (considering internal as well as international migration), these projections can help researchers and policymakers assess the future strain on critical resources and ecosystems, as well as the likely implications in terms of greenhouse gas emissions.

The value of population projections is also showcased by Engström and Kolk (this volume). Relying on the well-established IPAT framework, the authors project the future ecological impacts of population growth in terms of both climate change and land use. In line with the Debate contribution by Lutz (this volume), the authors conclude that the role of fertility decline in reducing global warming via emissions might be smaller than was previously assumed, particularly if the effects of ongoing population growth can be partly mitigated by the use of improved technology. They warn, however, that the effects of population growth on future land use are harder to mitigate through technological advancements, thus increasing the potential impact of contemporary fertility choices.

Projections can be used not only to assess humanity’s impact on the environment, but also to identify vulnerable populations and to derive estimates of the exposure of future populations to the likely impacts of climate change (Muttarak, 2021). As shown by Soltani et al. (this volume) for the case of Iran, population growth can lead to increasing vulnerability to water scarcity, primarily for urban residents. But projections have also been used to assess the impacts of sea level rise in coastal areas around the world, where much of the population growth in the 21st century is predicted to take place (Hauer et al., 2019). In both of these cases, studying the underlying demographic trends can aid in the development of targeted adaptation measures aimed at strengthening the resilience of potentially vulnerable populations. Incorporating demographic data into climate models further improves the accuracy of future emissions trajectories, and, in turn, the effectiveness of climate policies. By simulating different scenario assumptions, projections enable researchers to assess the potential consequences of different policy options and to make better-informed decisions.

This central idea is also enshrined in the design of the Shared Socio-Economic Pathways (SSPs) (O’Neill et al., 2017). The SSPs are scenario narratives used to project and analyze future global changes in the domains of technology, economy and demography, and their environmental impact. Population plays a crucial role in the SSPs driving several other major outputs, including, for example, economic development, urbanization, labor force participation, resource demand and consumption, and vulnerabilities and adaptive capacities with respect to climate change. Moreover, understanding population dynamics is key for designing effective climate policies, such as those aimed at mitigating emissions through behavioral changes or technology adoption. While the SSPs cannot resolve the general problem of the uncertainty of future projections, the five different pathways described by the SSPs can help researchers explore the implications of ongoing developments assuming different future intervention strategies. By incorporating detailed demographic projections, the SSPs provide a comprehensive framework for assessing how different pathways of population growth and distribution will affect global sustainability and climate change mitigation and adaptation efforts.

## Toward a holistic demographic perspective

Human and environmental systems are locked in a complex, dynamic and multi-dimensional relationship, whereby humans shape their environment and the environment shapes humans. Investigating and understanding the population-climate change linkages by looking at both the ways in which humans cause climate change and the ways in which climate change impacts human health, well-being and decision-making are core components of contemporary demographic science. Climate change impacts people’s daily lives, regardless of the speed or the geographic extent of the threats it poses. It influences livelihood strategies and childbearing plans, and has significant, long-lasting effects on individual behavior, migration decision-making and health and well-being outcomes.

A holistic demographic perspective is therefore essential for understanding the complex interactions between population and climate change, and for developing effective and equitable mitigation and adaptation strategies. As is shown by the articles included in this volume, investigating these linkages requires careful use of demographic data (often spatially detailed data) and associated demographic methods. Climate change investigations require population scientists to identify and quantify aspects of the environment across spatial and temporal scales that align with specific population processes or behaviors of interest. Thus, research on the climate change-population nexus is leading to advances in technical and data aspects of demographic sciences, while also pushing demographers to refine and refute foundational theories of population change and population-environment interactions (Grace and Merchant, 2022).

To meet this need for a comprehensive approach grounded in data, four points emerge from the literature published in this volume.

### Data and methodological advances

In their Debate article, Baschieri and Snow (this volume) argue for a more evidence-based use of demographic data to support climate mitigation and adaptation efforts within the framework of rights-based, people-centered population policies aimed at promoting climate action. As more data become available, demographers must adopt innovative models that can handle the complexity of climate-population interactions. These models should account for the spatio-temporal dimensions of climate impacts, such as delayed mental health effects and localized environmental stressors. Methodological innovations, including the use of big data, longitudinal studies and causal analyses, can enhance our understanding of these dynamics. Recent methodological advances in demography have also underscored the importance of spatial perspectives for understanding climate-population interactions. The availability of more granular spatial data has enabled researchers to conduct fine-grained analyses that capture local variations in climate impacts and population responses. This spatially nuanced approach allows for more accurate assessments of vulnerability and resilience at the community level to inform targeted interventions.

### Projections

Demographic projections have also evolved to incorporate complex interactions between population dynamics and environmental changes. Advances in computational power and modeling techniques have facilitated the development of sophisticated projection models that consider multiple variables and scenarios. These models and their improvements are critical for anticipating future population trends and their implications for resource demands, environmental stress and climate adaptation needs. At the same time, projection methods allow researchers to explicitly account for uncertainty regarding the future pathways of societies and demographic processes. As is pointed out by Lutz in his Debate article (this volume), future trends in both population and climate change do not always follow smooth trajectories toward ultimate stabilization, but are instead often characterized by unexpected and difficult-to-anticipate patterns.

### Interdisciplinary collaborations

The integration of interdisciplinary insights is crucial for gaining a holistic understanding of the climate-population nexus. Collaboration between demographers, climate scientists, economists, sociologists, geographers and public health experts enriches the analytical framework and enhances the robustness of the findings. Interdisciplinary research can provide comprehensive solutions that address the multifaceted challenges posed by climate change, and demographers are well-positioned to engage with interdisciplinary teams (Fussell et al., 2014). As Merchant and Grace observe in their Debate article (this volume), demography is in a unique position to address interdisciplinary research questions, as it is situated at the intersection of the natural and the social sciences, and it deals specifically with rates of change in social and natural processes.

### Complex justice questions

The differential impacts of climate change on various population subgroups raise complex justice questions. Vulnerable populations, including poor people, elderly people, children and marginalized communities, often bear the brunt of climate impacts despite contributing the least to greenhouse gas emissions. Addressing these inequities requires policies that promote social justice and inclusive development. Modeling efforts must incorporate justice and equity considerations to ensure that mitigation and adaptation strategies do not exacerbate existing inequalities (Zimm et al., 2024). In her Debate article (this volume), Muttarak argues that campaigning for reductions in population growth as a way to fight climate change will not address the root causes of the problem. Instead, a concerted effort is needed to bring down emissions, including by minimizing unsustainable consumption patterns and reducing reliance on fossil fuels, while ensuring that climate action is both equitable and just.

Finally, several priorities should be underlined in the study of climate and population. There is a need for more empirical evidence on the long-term impacts of climate change on demographic processes. Research of this kind must consider the heterogeneity in climate change impacts and responses, and develop modeling strategies that account for variability across scales. Additionally, the interactions between climate-induced migration and other demographic processes (including labor needs) and mitigation efforts require further exploration. Population aging in the context of climate change should also be investigated from the perspective of both population-level impacts and individual-level risks.

Future research should continue to develop holistic approaches that link different demographic events and changes under the umbrella of climate change. For instance, examining the interplay of health, migration and environmental stress can provide deeper insights into the multifaceted impacts of climate change. It is also crucial to identify the distinct mechanisms through which different climatic extremes affect demographic outcomes, as these mechanisms can differ significantly. The concepts of the Anthropocene and planetary boundaries (Richardson et al., 2023) can also be usefully integrated into demographic research to better understand broader environmental constraints and their implications for population dynamics.

The age of big data presents both opportunities and challenges for demographic research. Demographers must (continue to) embrace new data sources, such as satellite imagery and mobile phone or social media data, while addressing representativeness and data quality issues and ethical concerns. Improving analytical tools to enable them to integrate diverse data sources will facilitate more comprehensive and timely analyses of climate-population interactions. This also implies a call for more curricula in demography that incorporate modules on new data and analyses, as well as on key topics such as population displacement due to climate change and environmental disasters (Pesando et al., 2023).

Climate change has emerged as a defining challenge of the 21st century, necessitating a concerted effort by the demographic community to contribute to sustainable solutions. A holistic perspective, grounded in empirical evidence and interdisciplinary collaboration, is vital for understanding and addressing the complex interdependencies between population dynamics and the climate. The contributions to this special issue showcase the diversity of the approaches that have been applied and their usefulness in addressing pertinent climate research questions. The journey ahead requires sustained momentum, collaborative efforts and a commitment to integrating demographic knowledge into the broader climate change agenda.
